# Parkinson’s disease as a systemic pathology

**DOI:** 10.18632/aging.101824

**Published:** 2019-02-11

**Authors:** Mario Ezquerra, María-José Martí, Rubén Fernández-Santiago

**Affiliations:** 1 Laboratory of Neurodegenerative Disorders, Department of Neurology-Hospital Clínic of Barcelona, IDIBAPS, UB, 08036 Barcelona, Catalonia, Spain; 2 Centro de Investigación Biomédica en Red de Enfermedades Neurodegenerativas (CIBERNED), 28031 Madrid, Spain

**Keywords:** fibroblasts, Parkinson’s disease, transcriptomic

Parkinson’s disease (PD)is a progressive neurodegenerative disease characterized by the presence of proteinaceous aggregates containing α-synuclein (Lewy bodies and Lewy neurites), and neural loss of dopaminergic neurons (DAn) at the midbrain sustantia nigra, which has been associated with classical motor symptoms (tremor, rigidity and bradykinesia). Although the nigral DAn are the ultimate cells targeted by disease, there are also Lewy bodies in other extra-nigral neurons. The selective vulnerability of DAn has been related with the oxidative stress caused by the metabolism of dopamine, the large dendrite arborization requirements of these neurons in humans, and recently, with the presence of neuromelanin deposits as a consequence of the metabolism of dopamine. In addition, the presence of PD-associated biological alterations has been described not only in PD brains but also in peripheral autonomic nerves [1]. Moreover, recent studies have described presence of whole expression changes in non-nervous tissues like blood from PD patients and also its derivates [2]. Recently, we have found large global gene expression changes in primary cultures of fibroblasts from PD patients carrying mutations in the Parkin gene (*PRKN*) [3] thus supporting a novel view of PD as a systemic disease that can also affect peripheral non-neural tissues. A possible interpretation is that there could exist intrinsic alterations related with the disease in each individual cell of the skin and potentially in any cell of the body, i.e. transcriptomic alterations, that could be originated specifically by Parkin mutations and/or the environmental history of the subjects as mirrored in the fibroblast. Yet these changes would not cause any apparent skin or blood phenotype in PD patients, probably owing to the high rate of renewal of these cells, but possibly latent but sustained subclinical alterations in the skin.

The presence of transcriptomic alterations in cultured fibroblast has been described in other neurodegenerative diseases like amyotrophic lateral sclerosis (ALS) and primary lateral sclerosis [4]. Thus it is possible that the presence of systemic alterations in non-neural tissues could be a common hallmark of a variety of neuro-degenerative diseases as it has been previously suggested [5]. In addition, and taking into account the cell inaccessibility of DAn from living patients, primary fibroblast cultures obtained by skin biopsy from PD patients have been proposed as a cellular model to investigate molecular alterations in PD, mostly because their advantages including easy availability and robustness as cell system genuine to PD patients [5]. In our study the differentially expressed genes identified in fibroblast from PD patients with *PRKN* mutations were related with extracellular matrix organization, cell adhesion, and axon guidance pathways. Interestingly, the molecular processes involved in filopodia formation are largly common in fibroblast and neurons, ultimately leading in the later to the formation of axonal processes. In addition, we have also found deregulation of the same pathways in fibroblasts from both sporadic PD and LRRK2-associated PD (own unpublished findings). We have also previously found deregulation of these pathways at the transcriptome level in induced pluripotent stem cell (iPSC)-derived DAn from sporadic and LRRK2-associated PD patients which were generated upon cell reprogramming of somatic skin cells [6]. Again, independent studies also found enrichment of deregulated genes related with these cytoskeleton-associated pathways in postmortem dopa-minergic brain tissues from PD patients [7].

In summary, the large overlap of altered biological pathways detected in fibroblasts, iPSC-derived DAn, and postmortem brain tissues from PD patients support that fibroblasts could be a good surrogate cell model suitable to investigate the biological basal processes occurring in the brain from PD patients. In addition, our findings support the view of PD as a systemic disease in which molecular alterations can occur not only in the central and peripheral nervous system, but also in peripheral non-neural tissues such as the skin. 
